# Increased expression of neuropilin 1 in melanoma progression and its prognostic significance in patients with melanoma

**DOI:** 10.3892/mmr.2015.3752

**Published:** 2015-05-07

**Authors:** JING LU, YABIN CHENG, GUOHONG ZHANG, YUN TANG, ZIMING DONG, KEVIN J MCELWEE, GANG LI

**Affiliations:** 1 Department of Dermatology and Skin Science, Research Pavilion, Vancouver Coastal Health Research Institute, University of British Columbia, Vancouver, BC V5Z IL8, Canada; 2 Department of Pathophysiology, Basic Medical College, Zhengzhou University, Zhengzhou, Henan 450001, P.R. China; 3 Collaborative Innovation Center of Henan Province for Cancer Chemoprevention, Zhengzhou, Henan 450001, P.R. China; 4 Department of Pathology, Shantou University Medical College, Shantou, Guangdong 515000, P.R. China

**Keywords:** neuropilin 1, melanoma, prognosis, progression, matrix metalloproteinase 2

## Abstract

Neuropilin 1 (NRP1), a receptor of vascular endothelial growth factor (VEGF), promotes angiogenesis, tumor growth, tumor invasion and metastasis. However, the function of NRP1 in melanoma progression, as well as the effect of NRP1 expression on the prognosis of patients with melanoma remains unknown. In the present study, NRP1 expression was examined in 460 cases of melanocytic lesions (28 common nevi, 51 dysplastic nevi, 250 primary melanoma and 131 metastatic melanoma) at different stages, using a tissue microarray. The correlation of NRP1 expression with melanoma progression, and its prognostic value in patients with melanoma was examined. In addition, the correlation between matrix metalloproteinase 2 (MMP2) and NRP1 expression in patients with melanoma was analyzed. The results demonstrated that NRP1 expression was significantly increased in primary (56%) and metastatic melanoma (62%), compared with common nevi (11%) and dysplastic nevi (24%). Notably, increased NRP1 expression was correlated with a poorer overall, and disease specific, 10-year survival (P=0.03 and P=0.002, respectively). Multivariate Cox regression analyses indicated that NRP1 is an independent prognostic marker for melanoma. Furthermore, a significant positive correlation between NRP1 and MMP2 expression in melanoma biopsies was observed, and their concomitant expression was closely correlated with melanoma patient survival, further supporting the hypothesis that the expression of NRP1 is associated with melanoma invasion and metastasis. In conclusion, increased NRP1 expression is associated with disease progression and reduced survival in patients with melanoma, and is a promising prognostic molecular marker for this disease.

## Introduction

Melanoma is the cutaneous malignancy with the highest mortality and its incidence has continued to grow over the last 30 years (1). Although melanoma accounts for only 4% of all dermatological cancers, it is responsible for >80% of mortalities due to skin cancer and <10% of patients with metastatic melanoma survive for 5-years (2,3). The discovery and application of biomarkers, in conjunction with conventional cancer diagnosis, staging, and prognosis, may be useful in improving early diagnosis, screening and the subsequent management of these patients (4). However, at present, reliable markers are lacking and the prognosis of patients with melanoma remains poor. Therefore, an improved understanding of the regulatory factors contributing to melanoma initiation, progression and metastasis is required.

The neuropilins are multifunctional proteins that are involved in neural and vascular development, immunity and cancer (5). Neuropilins include two homologous proteins, NRP1 and NRP2, which are single-pass plasma membrane receptors, that were originally identified as binding to a tyrosine kinase receptor for semaphorin family members and vascular endothelial growth factor (VEGF). Recent evidence also suggests a role of neuropilins in cancer progression as a consequence of their interaction with VEGF (6). Furthermore, neuropilins have been observed to interact with platelet-derived growth factor (7) and other growth factors. These data support the hypothesis that neuropilins function as a signaling platform, regulating cancer cells and cells in the tumor microenvironment.

NRP1 is involved in angiogenesis, axon guidance, cell survival, invasion and migration (8). A number of types of malignant tumor cell express NRP1, and this appears to contribute to tumor cell aggressiveness (9,10). Furthermore, it has been demonstrated that a soluble form of NRP1 significantly inhibits VEGF-induced acute myeloid leukemia progression in a mouse model (11). Blocking NRP1 function produced a synergistic effect with that of anti VEGF, leading to inhibition of non-small-cell lung cancer (NSCLC) growth, which suggests that NRP1 may be a potential target for improving the efficacy of anti-VEGF therapy (12). A study also demonstrated that increased expression of NRP1 correlates with the growth and spread of medulloblastoma, and with poor survival in patients with medulloblastoma. In addition, placental growth factor acts through NRP1, rather than VEGF receptor 1, in order to promote tumor cell survival (13). Increased expression of the VEGF receptors (FLT1, KDR and NRP1) and of thrombospondin1 is associated with glomeruloid microvascular proliferation in malignant melanoma (14). Deletion of NRP1 in healthy epidermis prevents skin tumor initiation (15). The results of a recent study add to the evidence suggesting that NRP1 expression promotes invasiveness of melanoma cells through VEGFR2-dependent and -independent mechanisms (16). However, the significance of NRP1 in melanoma progression, diagnosis and prognosis remains unknown.

Matrix metalloproteinase 2 (MMP2) is a zinc-dependent proteinase that is capable of cleaving extracellular matrix substrates. Degradation of the matrix is a crucial event in the progression, invasion and metastasis of cancer cells. Increased MMP2 expression was shown to predict adverse outcomes in patients with breast cancers (17). Recent studies have also shown that MMP2 is inversely correlated with the survival of patients with melanoma (18,19). It has been demonstrated that MMP2 is closely correlated with VEGF signaling in cancer cell growth, invasion and metastasis (20,21). NRP1 is an important receptor for VEGF. Therefore, the present study further examined the correlation between NRP1 and MMP2 expression in melanoma biopsies, and analyzed the combined effect of NRP1 and MMP2 expression in predicting patient outcomes.

## Materials and methods

### Ethics statement

The use of human skin tissues was approved by the Clinical Research Ethics Board of the University of British Columbia (Vancouver, BC, Canada; UBC CREB number: H09-01321) (22). The present study was conducted according to the principles expressed in the Declaration of Helsinki.

### Tissue microarray (TMA) construction

**Figure 1:**
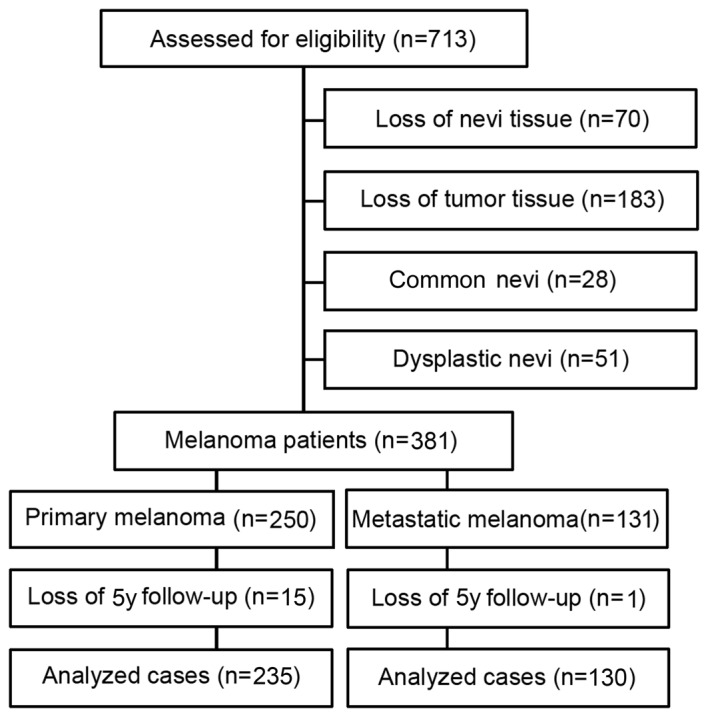
Diagram of patient inclusion and exclusion.

The collection of melanoma specimens and the construction of the tissue microarray (TMA) have been previously described (22,23). Briefly, formalin-fixed, paraffin-embedded tissues from 49 common nevi, 100 dysplastic nevi, 402 primary melanomas and 162 metastatic melanomas were used for the TMA construction. All specimens were obtained from the 1990–2009 archives of the Department of Pathology, Vancouver General Hospital (Vancouver, BC, Canada) (22,23). The most representative tumor area was selected and marked on the hematoxylin and eosin stained slides, and the TMAs were assembled using a tissue-array instrument (Beecher Instruments, Silver Spring, MD, USA). Due to the loss of biopsy cores or insufficient tumor cells present in the cores, 70 nevi and 183 melanomas were excluded from analysis. Therefore, 28 common nevi, 51 dysplastic nevi, 250 primary melanomas and 131 metastatic melanomas were evaluated for NRP1 staining (Fig. 1).

### Immunohistochemistry of TMA

Immunohistochemistry was performed as described previously (22,23). TMA slides were dewaxed at 55°C for 30 min and then washed with xylene (Thermo Fisher Scientific, Waltham, MA, USA). Tissues were rehydrated by a series of washes in 100, 95 and 80% ethanol, followed by two washes in distilled water. Antigen retrieval was performed by heating the samples at 95°C for 30 min in 10 mmol/l sodium citrate (pH 6.0; Sigma-Aldrich, St. Louis, MO, USA). After inactivating the endogenous peroxidase by incubating in 3% H_2_O_2_ (Sigma-Aldrich) for 30 min and blocking with universal blocking serum for 30 min, slides were incubated with a primary mouse monoclonal anti-NRP1 antibody (1:25; cat. no. sc-5307; Santa Cruz Biotechnology, Inc., Santa Cruz, CA, USA) at 4°C overnight. Negative controls were produced by omitting the NRP1 antibody during the primary antibody incubation step. The slides were then incubated with a biotinylated streptavidin conjugated horseradish peroxidase anti mouse and anti-rabbit universal secondary antibody (cat. no. KO609; DAKO Diagnostics, Glostrup, Denmark) for 30 min each, followed by developing with a diaminobenzidine substrate kit (DAKO Diagnostics) and counterstaining with hematoxylin.

### Evaluation of immunostaining

Positive NRP1 immunostaining was defined as cytoplasmic and membrane staining, and graded according to the intensity and percentage of cells with positive staining. The evaluation of NRP1 staining was done microscopic examination of the tissue sections by two observers (including one pathologist), who were blinded to the status of the samples, using a microscope (Olympus BX40; Olympus, Tokyo, Japan). NRP1 staining intensity was scored as 0, 1+, 2+ and 3+. The percentage of NRP1-positive cells in the samples was also assigned to one of four categories: 1, 0–25%; 2, 26–50%; 3, 51–75%; and 4, 76–100%. On the basis of the immunoreactive score, the staining pattern was defined as: Negative, (0); weak, (1–4); moderate, (6–8); or strong, (9–12). The optimal cut-off points for the staining score were calculated using the MedCalc software for Windows, version 12.5 (MedCalc Software, Ostend, Belgium). The best area under the ROC curve (AUC) was used to determine the optimal cut-off point of staining. Based on the AUC value, the optimal cutoff point for the NRP1 staining was identified as 4. The staining pattern of the biopsies was defined as: 0–4, low and 6–12, high. The correlation between NRP1 and MMP2 expression was examined in 365 cases (234 primary and 131 metastatic melanoma).

### Statistical analysis

Differences in the demographic and clinical characteristics, and in NRP1 expression between patient subgroups were evaluated by the Kruskal-Wallis test and χ^2^ tests. Survival time was calculated from the date of melanoma diagnosis to the date of death or of the last follow-up. The effect of NRP1 expression on the overall and disease-specific survival was evaluated using Kaplan-Meier analysis and a log-rank test. Univariate and multivariate Cox proportional hazard regression models were performed in order to estimate the hazard ratios (HRs) or adjusted HRs, and their associated 95% confidential intervals (CIs). P<0.05 was considered to indicate a statistically significant difference. SPSS version 16 (SPSS Inc., Chicago, IL, USA) software was used for all analyses.

## Results

### NRP1 expression is positively correlated with melanoma progression

**Figure 2:**
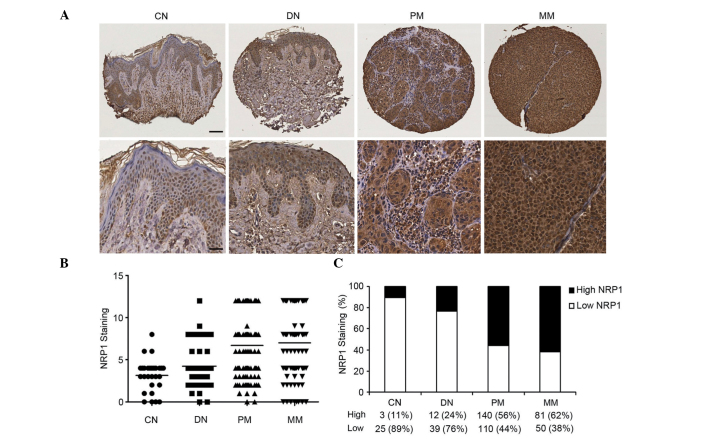
Increased NRP1 expression is correlated with melanoma progression. (A) Representative images of CN and DN, with low NRP1 expression, and PM and MM, with high NRP1 expression (upper panel, scale bar 40 *µ*m; lower panel, scale bar 20 *µ*m). (B) Kruskal-Wallis test for differences in NRP1 staining among CN, DN, PM and MM. The mean is depicted as a horizontal line in each group (n=460, P<0.0001). (C) NRP1 expression was increased from CN to DN, PM and MM (*n*=460, P=3.6×10^−9^, χ^2^ test). Magnification, ×100 (upper panel), ×200 (lower panel). NRP1, neuropilin 1; CN, common nevi; DN, dysplastic nevi; PM, primary melanoma; MM, metastatic melanoma.

NRP1 staining was stronger in primary and metastatic melanoma biopsies than that in common nevi and dysplastic nevi cases (Fig. 2A). Kruskal-Wallis test on the NRP1 scoring pattern in the patient samples revealed that NRP1 expression increased significantly from common nevi (mean 3.1) and dysplastic nevi (mean 4.2), to primary melanoma (mean 6.7) and metastatic melanoma (mean 70; P<0.0001, CN+DN vs. PM+MM; Fig. 2B). Furthermore, χ^2^ test revealed that the percentage of high NRP1 staining was significantly greater in primary melanoma (56%) and metastatic melanoma (62%), compared with that in common nevi (11%) and dysplastic nevi (24%) (P=3.6×10^−9^, CN+DN vs. PM+MM; Fig. 2C).

### NRP1 expression is positively correlated with American Joint Committee on Cancer (AJCC) stage, tumor thickness and ulceration

**Figure 3:**
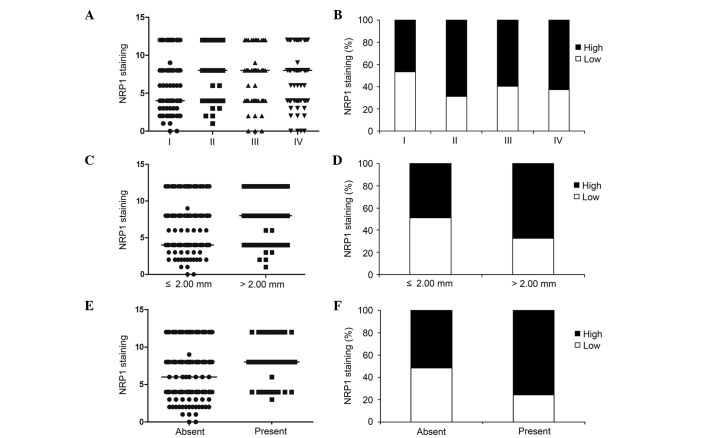
NRP1 expression correlates with melanoma AJCC stage, tumor thickness and ulceration. (A) Kruskal-Wallis test for differences in NRP1 staining among AJCC stages I–IV. The median is depicted as a horizontal line in each group (n=377, P=0.007). (B) Difference in NRP1 expression among AJCC stages I–IV (n=377, P=0.003, χ^2^ test). Melanomas in AJCC stages II, III and IV exhibited a higher percentage of high NRP1 expression compared with melanomas in stage I (n=377, P=0.0005, χ^2^ test). (C) Differences in NRP1 staining between melanoma thickness ≤2.0 mm and >2.0 mm. The median is depicted as a horizontal line in each group (n=250, P=0.007, t-test). (D) Melanomas >2.0 mm exhibited a higher percentage of high NRP1 expression compared with melanomas ≤2.0 mm (n=250, P=0.004, χ^2^ test). (E) Differences in NRP1 staining between melanoma patients with no ulceration and with ulceration. The median is depicted as a horizontal line in each group (n=250, P=0.004, t test). (F) Increased NRP1 expression was correlated with ulceration of melanomas (n=250, P=0.004, χ^2^ test). NRP1, neuropilin 1; AJCC, American Joint Committee on Cancer.

As NRP1 expression was correlated with melanoma progression, the correlation between NRP1 expression and various clinicopathological characteristics was also investigated. Kruskal-Wallis test on the NRP1 scoring pattern in the melanoma samples revealed that NRP1 expression increased significantly from AJCC I (median 4) to AJCC II–IV (median 8; (P=0.007, Fig. 3A). As shown in Fig. 3B and Table I, high expression of NRP1 was detected in 47% of melanoma specimens at AJCC stage I compared with 60–69% of melanoma specimens at AJCC II–IV (P=0.0005). However, no significant difference was found in NRP1 expression among AJCC stages II–IV, indicating that increased No significant difference of NRP1 expression was found among AJCC stages II to IV, whereas a significant increase was detected between stage I and II (P=0.0004, stage I vs. stage II). In primary melanoma, NRP1 expression was increased in tumors with a thickness >2.00 mm (median 8), compared with melanomas with a thickness ≤2.00 mm (median 4; P=0.007, Fig. 3C). Furthermore, high NRP1 expression was observed in 67% of melanomas with a thickness >2.00 mm, compared with 49% of tumors with a thickness ≤2.00 mm (P=0.004; Fig. 3D, Table I). In addition, NRP1 expression was higher in melanomas with ulceration (median 8), compared with that in melanomas with no ulceration (median 6; P=0.004; Fig. 3E), which is in accordance with the χ^2^ test results, indicating that high NRP1 expression was observed in 76% of melanomas with ulceration, compared with 52% of melanomas with no ulceration (P=0.004; Fig. 3F, Table I).

### Increased NRP1 expression is associated with poor survival in patients with melanoma

**Figure 4:**
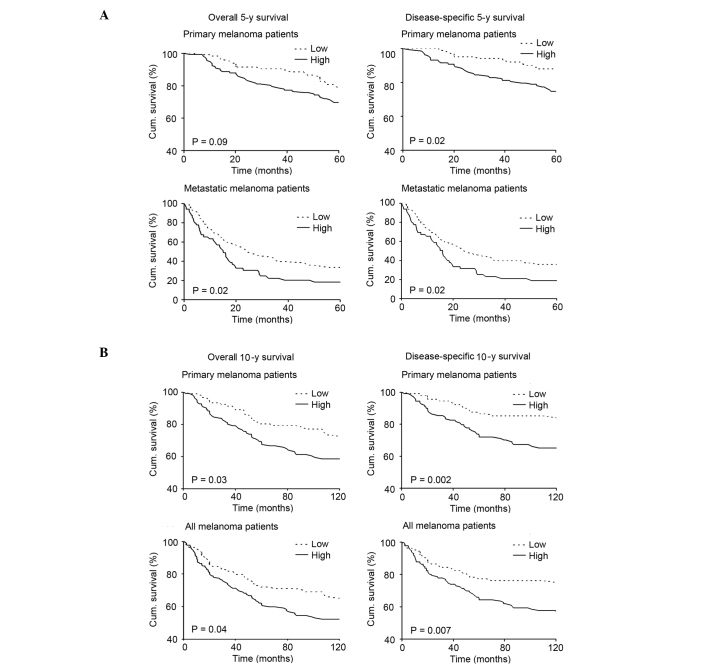
Kaplan-Meier analysis of the correlation between NRP1 expression, and 5-year survival and 10-year survival. (A) Upper lane: Overall and disease-specific 5-year survival of patients with primary melanoma (n=235; P=0.09 and P=0.02 respectively; log-rank test). Lower lane: Overall and disease-specific 5-year survival of patients with metastatic melanoma (n=130; P=0.02 for each; log-rank test). Labels at the top of the figure apply to all graphs in the same column. (B) Upper lane: Overall and disease-specific 10-year survival of patients with primary melanoma (n=202; P=0.03 and P=0.002 respectively; log-rank test). Lower lane: Overall and disease-specific 10-year survival of all melanoma patients (n=235; P=0.04 and P=0.007 respectively; log-rank test). Labels at the top of the figure apply to all graphs in the same column. NRP1, neuropilin 1.

In order to investigate whether NRP1 expression is associated with 5-year survival of patients with melanoma at specific stages of the disease, the patient cohort was divided into those with primary melanoma and those with metastatic melanoma, and patient survival in each group was analyzed. The Kaplan-Meier survival curve revealed that patients with primary or metastatic melanoma who exhibited low NRP1 expression had a better overall 5-year survival than patients with high NRP1 expression (P=0.09 and 0.02, respectively; Fig. 4A, left column). Furthermore, patients with low NRP1 expression who had a diagnosis of primary or metastatic melanoma had a significantly better disease-specific 5-year survival compared with patients with high NRP1 expression (P=0.02 for primary and metastatic groups; Fig. 4A, right column). In addition, the present study investigated whether NRP1 expression was associated with 10-year survival in patients with melanoma. As the number of patients with metastatic melanoma is relatively small for 10-year survival analysis, the 202 patients with primary melanoma and 235 patients with all stages of melanoma were analyzed. The results showed that patients with primary or all melanoma who had low NRP1 expression had a better overall 10-year survival than patients with high NRP1 expression (P=0.03 and 0.04, respectively; Fig. 4B, left column). Patients with low NRP1 expression, with primary or all melanoma also had significantly better disease-specific 10-year survival compared with patients with high NRP1 expression (P=0.002 and 0.007, respectively; Fig. 4B, right column). The results of Kaplan-Meier analysis were further confirmed by a Univariate Cox proportional hazards regression model for 10-year survival of patients with primary melanoma (HR, 1.7; 95% CI, 1.06–2.80; P=0.03, for overall survival; HR, 2.51; 95% CI, 1.36–4.64; P=0.003, for disease-specific survival; Table II).

### NRP1 expression is an independent prognostic marker for melanoma

The current study also examined whether NRP1 expression is an independent prognostic marker for survival of patients with melanoma patients, using multivariate Cox proportional hazard analysis. For 10-year survival, as the number of metastatic melanoma cases is relatively small, initial analysis was conducted in primary melanoma patients, adjusted for important clinical variables, such as age, thickness and ulceration. The results clearly indicate that, as with tumor thickness and the presence of ulceration, which have been widely accepted as independent prognostic factors for survival in patients with melanoma (24), NRP1 expression is an independent prognostic factor for overall (HR, 0.58; 95% CI, 0.35–0.99; P=0.04), and disease-specific 10-year survival (HR, 0.4.7; 95% CI, 0.25–0.89; P=0.02; Table II). Furthermore, multivariate Cox regression analysis of 5-year survival of 130 patients with metastatic melanoma was analyzed. The results showed that NRP1 expression was also correlated with overall (HR, 1.65; 95% CI, 1.07–2.54; P=0.02), and disease specific 5-year survival (HR, 1.64; 95% CI, 1.06–2.53; P=0.03) in patients with metastatic melanoma (Table III).

### NRP1 expression is positively correlated with MMP2 expression, and their concomitant expression is associated with reduced survival in patients with melanoma

**Figure 5:**
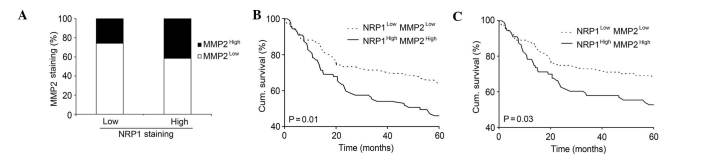
Correlation between NRP1 and MMP2 expression in melanoma. (A) Positive correlation between NRP1 and MMP2 expression in melanoma. Melanomas which had high NRP1 expression also exhibited a significantly higher percentage of high MMP2 staining (n=365; P=0.02; χ^2^ test). (B) and (C) Kaplan-Meier analysis of the correlation between concomitant NRP1 and MMP2 expression and overall (B) or disease-specific (C) 5-year survival of patients with melanoma (n=201; P=0.01 and P=0.03 respectively; log-rank test). NRP1, neuropilin 1; MMP2, matrix metalloproteinase 2.

MMP2 has been shown to be associated with increased invasion and poorer patient survival (18,19). As the TMA used for NRP1 staining in the present study was the same as as that previously used by this group to detect MMP2 (18), it was possible to analyze the correlation between NRP1 and MMP2 expression. The results demonstrated that high NRP1 was positively correlated with high MMP2 expression (P=0.02; Fig. 5A). The effect of combined NRP1 and MMP2 expression on patient survival was subsequently analyzed using Kaplan-Meier survival curves, and it was found that patients with low NRP1 as well as low MMP2 expression exhibited a significantly increased 5-year overall survival and disease specific survival, compared with patients with high NRP1 and high MMP2 expression (P=0.01 and 0.03, respectively. Fig. 5B,C). These data demonstrated that the concomitant expression of NRP1 and MMP2 exerts a significant influence on the survival of patients with melanoma.

## Discussion

Increased NRP1 expression has been detected in tumor cell lines and tumor biopsies of various origins (10,25–27). Furthermore, NRP1 expression correlates with more aggressive tumor behavior. For example, in breast cancer biopsies NRP1 expression is a feature of high-grade tumors, and is frequently expressed in tumors from patients who do not subsequently survive as a result of their cancer (28). In the present study, TMA technology and immunohistochemistry were used to investigate NRP1 expression in 460 cases of pigmented skin lesions at different stages. To the best of our knowledge, this is the first study to analyze the correlation between NRP1 expression, and melanoma progression and patient survival.

The results showed that NRP1 expression was significantly reduced in common nevi and dysplastic nevi, compared with primary melanoma and metastatic melanoma. This indicated that increased NRP1 activity may be a common requirement for the transformation from benign neoplasia to malignancy, as well as for tumor progression from primary to metastatic melanoma. This finding supports that of a separate study, which showed that deletion of NRP1 in normal epidermis prevents skin tumor initiation (15). The present study also found that NRP1 expression was positively correlated with the depth of tumor invasion (thickness ≤2.00) and ulceration of primary melanoma lesions, which is in accordance with other studies, showing that overexpression of NRP1 is correlated with tumor growth and metastasis in other types of cancer, thereby influencing tumor progression (25,27).

By constructing Kaplan Meier survival curves, it was shown that increased NRP1 expression was correlated with poor overall, and disease-specific 5-year survival in patients with primary and metastatic melanoma, and was correlated with poorer overall, and disease-specific 10-year survival in all melanoma patients. These correlations were further confirmed by univariate Cox regression analyses. Furthermore, multivariate Cox proportional hazard analysis also indicated that NRP1 expression is an independent prognostic marker for melanoma. These findings, regarding the function of NRP1 in melanoma, are in accordance with a previous study, indicating that NRP1 is an enhancer of cancer invasion, that patients with high expression of NRP1 have shorter disease-free and overall survival, and that NRP1 is an independent predictor of cancer relapse and poor survival in patients with NSCLC (10).

NRP1 is a specific co-receptor for the secreted VEGF-A165 isoform. VEGF mediates tumor angiogenesis and directly enhances tumor growth via VEGF/VEGFR autocrine loops. NRP1 forms complexes with Flk-1/KDR (VEGFR2) to enhance the binding of VEGF165 to VEGFRs, and promotes VEGF165-mediated tumor angiogenesis, cell migration and tumorigenicity (29,30). In a preclinical xenograft NSCLC model, administration of a function-blocking anti-NRP1^B^ antibody in order to block VEGF binding to NRP1, resulted in marginal tumor growth delay and additive effects to anti-VEGF therapy in reducing tumor growth. Further, tumor vascular density is decreased when anti-NRP1^B^ is combined with murine anti-VEGF (12), which may, therefore, make NRP1 a potential target for improving the efficacy of anti-VEGF therapy. Anti-NRP1, a novel antiangiogenesis agent, has been used in two phase I trials in patients with metastatic breast cancer (31).

Increasing evidence suggests an important role for MMPs, a large family of secreted peptidases, in tumor invasion and metastasis (32). MMP2, or gelatinase A, which digests primarily type IV collagen, is hypothesized to be involved in melanoma progression (33). Increased MMP2 expression has been shown to predict adverse outcomes in patients with breast cancer (17). Recent studies have also shown that MMP2 is associated with the survival of patients with melanoma (18,19). Furthermore, it has been shown that MMP2 expression is closely correlated with VEGF signaling in cancer cell growth, invasion and metastasis (20,21). For example, estrogen may increase the expression of VEGF, and thus activate the ERK1/2 pathway to induce MMP2/9 expression (20). In addition, MMP2 is involved in the autocrine regulation of VEGF A expression in melanoma cells (21).

NRP1 is known to be an important receptor for VEGF. Therefore, in the present study, greater emphasis was placed on elucidating the correlation between NRP1 and MMP2 expression (34). The results showed that in 365 melanoma samples, melanomas with high NRP1 expression also exhibited a significantly higher percentage of high MMP2 staining. Furthermore, patients with low NRP1 as well as low MMP2 expression had better overall and disease-specific 5-year survival compared with patients who exhibited high NRP1 and high MMP2 expression. Based on this results, it is hypothesized that a powerful cell survival regulator, such as NRP1, may be a positive regulator of MMP2, and therefore promote melanoma progression, invasion and metastasis.

In conclusion, the current study demonstrated that NRP1 expression is significantly correlated with the progression of human melanoma. Notably, high NRP1 expression was correlated with a poorer 5-year and 10-year survival in patients with melanoma, and was shown to be an independent prognostic factor. Furthermore, there was a significant positive correlation between NRP1 and MMP2 expression in melanoma biopsies, and their concomitant expression was inversely correlated with the survival of patients with melanoma. These data suggest that NRP1 is involved in melanoma pathogenesis and that it may serve as a prognostic marker for patients with this disease.

## Figures and Tables

**Table I:** NRP1 staining and clinicopathologic characteristics of 381 melanomas.

Variables	NRP1 staining		Total	P value
	Low	High		
All melanoma (n=381)				
Age, years				
≤60	95 (47.0)	107 (53.0)	202 (53.0)	0.03
>60	65 (36.3)	114 (63.7)	179 (47.0)	
Gender				
Male	88 (38.1)	143 (61.9)	231 (60.6)	0.06
Female	72 (48.0)	78 (52.0)	150 (39.4)	
AJCC stage				
I	77 (53.5)	67 (46.5)	144 (38.2)	0.003^a^
II	33 (31.1)	73 (68.9)	106 (28.1)	0.0005^b^
III	21 (40.4)	31 (59.6)	52 (13.8)	
IV	28 (37.3)	47 (62.7)	75 (19.9)	
Primary melanoma (n=250)				
Age, years				
≤60	58 (46.8)	66 (53.2)	124 (49.6)	0.38
>60	52 (41.3)	74 (58.7)	126 (50.4)	
Gender				
Male	56 (40.6)	82 (59.4)	138 (55.2)	0.23
Female	54 (48.2)	58 (51.8)	112 (44.8)	
Tumor thickness (mm)				
≤2	78 (51.3)	74 (48.7)	152 (60.8)	0.004
>2	32 (32.6)	66 (67.4)	98 (39.2)	
Ulceration				
Absent	99 (48.3)	106 (51.7)	205 (82.0)	0.004
Present	11 (24.4)	34 (75.6)	45 (18.0)	
Subtype				
Lentigo maligna	24 (47.1)	27 (52.9)	51 (22.0)	0.29
Superficial spreading	46 (50.0)	46 (50.0)	92 (39.7)	
Nodular	14 (32.6)	29 (67.4)	43 (18.5)	
Unspecified	20 (43.5)	26 (56.5)	46 (19.8)	
Site^c^				
Sun protected	82 (45.1)	100 (54.9)	182 (72.8)	0.58
Sun exposed	28 (41.2)	40 (58.8)	68 (27.2)	
Metastatic melanoma (n=131)				
Age, years				
≤60	35 (41.7)	49 (58.3)	78 (59.5)	0.17
>60	18 (25.4)	41 (74.6)	53 (40.5)	
Gender				
Male	32 (34.4)	61 (65.6)	93 (71.0)	0.17
Female	18 (47.4)	20 (52.6)	38 (29.0)	

a Comparison among AJCC stages I IV, χ^2^ test.

b Comparison between AJCC stage I and stages II–IV, χ^2^ test.

c Sun-protected sites: trunk, arm, leg and feet; sun-exposed sites: head and neck. Data are presented as number of cases (percentage) AJCC, American Joint Committee on Cancer.

**Table II:** Univariate and multivariate Cox regression analysis of 10-year survival of 202 patients with primary melanoma.

A, Overall survival										
Variable	Univariate Cox regression				P	Multivariate Cox regression				P
	β^a^	SE	HR	95% CI		β^a^	SE	HR	95% CI	
Age	−1.22	0.26	0.29	0.18–0.49	4×10^−6^	−0.79	0.28	0.45	0.26–0.79	0.005
Thickness	1.64	0.27	5.14	3.03–8.71	1×10^−9^	−1.31	0.32	0.27	0.14–0.50	4×10^−5^
Ulceration	1.41	0.25	4.10	2.50–6.73	2×10^−8^	−0.67	0.27	0.51	0.30–0.87	0.01
NRP1	0.54	0.25	1.72	1.06–2.80	0.03	−0.54	0.27	0.58	0.35–0.99	0.04

B, Disease-specific survival										
Variable	Univariate Cox regression				P	Multivariate Cox regression				P
	β^a^	SE	HR	95% CI		β^a^	SE	HR	95% CI	
Age	−0.83	0.29	0.44	0.25–0.77	0.004	−0.41	0.31	0.67	0.36–1.22	0.19
Thickness	1.82	0.33	6.15	3.22–11.75	4×10^−8^	−1.42	0.37	0.24	0.12–0.50	1×10^−4^
Ulceration	1.51	0.29	4.52	2.56–7.99	2×10^−7^	−0.77	0.31	0.46	0.25–0.85	0.01
NRP1	0.92	0.31	2.51	1.36–4.64	0.003	−0.76	0.33	0.47	0.25–0.89	0.02

a β, regression coefficient. Age was coded as 1 (≤60 years) and 2 (>60 years) for all melanoma. Thickness was coded as 1 (≤2.00 mm) and 2 (>2.00 mm). Ulceration was coded as 1 (absent) and 2 (present). Low NRP1 staining was coded as 1 and high NRP1 staining was coded as 2. SE, standard error; HR, hazard ratio; CI, confidence interval; P, P-Value; NRP1, neuropilin 1.

**Table III:** Multivariate Cox regression analysis of 5-year survival of 130 patients with metastatic melanoma.

Variable	Overall survival				P	Disease-specific survival				P
	β^a^	SE	HR	95% CI		β^a^	SE	HR	95% CI	
Age	−0.12	0.22	0.88	0.58–1.36	0.57	−0.09	0.22	0.91	0.59–1.41	0.68
Sex	−0.08	0.23	0.93	0.59–1.45	0.74	−0.12	0.23	0.89	0.56–1.39	0.60
NRP1	0.50	0.22	1.65	1.07–2.54	0.02	0.49	0.22	1.64	1.06–2.53	0.03

a β, regression coefficient. Age was coded as 1 (≤60 years) and 2 (>60 years). Sex was coded as 1 (male) and 2 (female). Low NRP1 staining was coded as 1 and high NRP1 staining was coded as 2. SE, standard error; HR, hazard ratio; CI, confidence interval; P, P-Value; NRP1, neuropilin 1.
